# Financial Hardship and Psychological Distress During the Pandemic: A Nationally Representative Survey of Major Racial-Ethnic Groups in the United States

**DOI:** 10.1089/heq.2022.0197

**Published:** 2023-07-20

**Authors:** Alia Alhomsi, Paula D. Strassle, Stephanie Ponce, Izzy Mendez, Stephanie M. Quintero, Miciah Wilkerson, Anita L. Stewart, Anna M. Napoles

**Affiliations:** ^1^ Division of Intramural Research, National Institute on Minority Health and Health Disparities, National Institute of Health, Bethesda, Maryland, USA.; ^2^ Center for Aging in Diverse Communities, Institute for Health and Aging, University of California San Francisco, San Francisco, California, USA.

**Keywords:** financial hardship, lost income, psychological distress, depression, anxiety, loneliness

## Abstract

**Introduction:**

While financial hardship has been consistently linked to psychological distress, little research exists on associations between financial hardship experienced during the pandemic and mental health.

**Methods:**

We conducted a nationally representative, online survey of American Indian/Alaska Native, Asian, Black/African American, Latino (English and Spanish speaking), Native Hawaiian/Pacific Islander, White, and multiracial adults, 12/2020–2/2021 (*n*=5500). Six financial hardship domains were measured (lost income, debt, unmet expenses, unmet health care expenses, housing insecurity, and food insecurity). Psychological distress measures included anxiety-depression symptoms (Patient Health Questionnaire-4), perceived stress (modified Perceived Stress Scale), and loneliness-isolation (“In the past month, how often have you felt lonely and isolated?”). Associations between financial hardship and psychological distress were estimated using multinomial logistic regression.

**Results:**

Overall, 70.3% of participants reported experiencing financial hardship (substantial hardship: 21.3%; some hardship: 27.4%; and a little hardship: 21.6%), with Spanish-speaking Latino (87.3%) and Native Hawaiian/Pacific Islander (79.2%) adults being most likely. Debt (57.6%), lost income (44.5%), and unmet expenses (33.7%) were the most common. There was a dose–response association between financial hardship and moderate/severe anxiety-depression symptoms (a little hardship: adjusted odds ratio [aOR]=1.42, 95% confidence interval [CI]=1.12–1.80; some hardship: aOR=3.21, 95% CI=2.58–3.98; and substantial hardship: aOR=8.15, 95% CI=6.45–10.29). Similar dose–response trends were observed with perceived stress and loneliness-isolation. No racial-ethnic difference in the association between financial hardship during the pandemic and psychological distress was seen.

**Discussion:**

Financial hardship has had a major impact on psychological distress during the pandemic; moreover, while no racial-ethnic difference in the effect of financial hardship was observed, because racial-ethnic minorities experienced greater hardship, financial hardship may exacerbate psychological distress disparities.

## Introduction

Aspects of psychological distress, such as depression, anxiety, stress, and loneliness, have been increasingly recognized as major public health concerns. Multiple factors may contribute to the development of psychological distress, including socioeconomic instability and global incidents like the coronavirus disease 2019 (COVID-19) pandemic.^1,2^ In the United States, COVID-19 mitigation protocols led to increased social isolation, and have amplified feelings of loneliness, stress, anxiety, and depressive symptoms. For example, in June 2020, 31% of adults reported symptoms of anxiety or depression,^3^ and in October 2020, 36% of all U.S. adults reported experiencing severe loneliness.^4^

The impacts of COVID-19 on psychological distress have also disproportionately affected several historically marginalized racial-ethnic populations, including Latino and Black/African American individuals.^3,5,6^ Latino and Black/African American adults have also been found to be at an increased risk of increased substance use and suicidal ideation during the pandemic, compared to White adults.^3^

Before the pandemic, financial hardship has been consistently linked to increased risk of psychological distress and poor mental health.^7–9^ The COVID-19 pandemic has had a profound financial impact on individuals living in the United States; however, Black/African American and Latino households have been shown to be more likely to experience financial hardship during the pandemic due to pre-existing disparities in socioeconomic status and income inequality.^10^ Current studies investigating the impact of experiencing financial hardship during the COVID-19 pandemic on psychological distress are limited, both in their ability to capture the complexity of financial hardship and the lack of inclusion of racially diverse populations.

To date, no study has included American Indian/Alaska Native, Native Hawaiian/Pacific Islander, or multiracial adults, and few have been nationally representative. Thus, the purpose of this study was to estimate the associations between financial hardship experienced during the pandemic, captured across six separate domains (lost income, unmet expenses, debt, unmet health care expenses, housing insecurity, and food insecurity), on psychological distress (anxiety-depression symptoms, perceived stress, and loneliness-isolation), and whether the association between financial hardship and psychological distress varies across race-ethnicity, using a nationally representative cohort of diverse adults living in the United States.

## Methods

We used the COVID-19's Unequal Racial Burden (CURB) survey, an online survey administered by YouGov, which uses a proprietary, opt-in survey panel comprising over 1.8 million U.S. residents, to conduct nationally representative online surveys. Panel members are recruited through a variety of methods to ensure diversity, and then matched to a theoretical target sample for each survey.

For this study, the target sample was drawn from the 2018 American Community Survey 1-year sample data, and included 1000 Asian, 1000 Black/African American, 1000 Latino (half of the surveys were conducted in Spanish), 1000 White, 500 American Indian/Alaska Native, 500 Native Hawaiian/Pacific Islander, and 500 multiracial adults ≥18 years of age (*n*=5500 total). Matched panel members who completed the online survey were then weighed to obtain nationally representative cohorts within each racial-ethnic group (e.g., Asian participants were weighted to represent all Asians living in the United States). Survey response rate was 20.0%. Details about the CURB survey development, sampling design, and participant demographics have been published previously.^11^

Surveys were completed online between December 8, 2020, and February 17, 2021. The National Institutes of Health Office of Institutional Review Board Operations determined that this study does not qualify as human subject research because data were de-identified (IRB# 000166).

We measured three aspects of psychological distress: anxiety-depression symptoms, perceived stress, and loneliness-isolation. A more in-depth description for all three of the psychological distress measures in the CURB survey has been described elsewhere.^12^

Anxiety-depression symptoms were measured using the Patient Health Questionnaire for Depression and Anxiety (PHQ-4), which asks about being bothered by feeling nervous/anxious, not able to control worrying, little interest or pleasure, and feeling depressed or hopeless in the past 2 weeks.^13^ All four questions had excellent internal reliability within our sample (Cronbach's alpha=0.91). Using the validated scoring of the PHQ-4, anxiety-depression symptoms were categorized as normal (0–2), mild (3–5), moderate (6–8), and severe (9–12); due to relatively low prevalence of severe symptoms in our cohort, a combined moderate/severe category was created.

Perceived stress was measured using the six negatively worded questions from the Perceived Stress Scale-10 (PSS-10), which ask about stress over the past month.^14^ Included questions asked about if they felt unable to control things and if they were stressed/nervous, could not cope, were upset by unexpected events, were angered by things they are unable to control, or could not overcome difficulties (response choices: never (1), almost never (2), sometimes (3), fairly often (4), and very often (5)). The modified PSS also had excellent internal reliability (Cronbach's alpha=0.94). Item responses were averaged and categorized as low (1 to <2), mild (2 to <3), moderate (3 to <4), and severe (≥4) stress. Similar to anxiety-depression symptoms, moderate and severe were combined due to relatively low prevalence of severe stress.

Loneliness-isolation was measured using a new, single question framed similar to the PSS-10 questions: “In the past month, how often have you felt lonely and isolated?” Responses included the following: never (0), almost never (1), sometimes (2), fairly often (3), and very often (4). For analyses, responses were collapsed into never (1), almost never/sometimes (2–3), and fairly often/very often (4–5).

Participants were also asked twelve questions relating to economic and financial hardship during the pandemic, the majority of which were adapted from the All of Us COVID-19 Participant Experience (COPE) survey. Participants were asked if there was a time during the pandemic when they did not have enough money to meet daily needs, pay monthly bills, pay for health care they needed, pay for medications, and pay rent, mortgage, or other housing costs.

Participants were also asked if there was a time when they were hungry but did not eat because there was not enough money for food, if there was a time when they did not have a regular place to live, as well as several questions about employment changes due to the pandemic (hours reduced, lost job), lost income during the pandemic, lost savings during the pandemic, increased debt during the pandemic, and lost health insurance during the pandemic. These questions were then categorized into six domains: (1) lost income, (2) debt, (3) unmet expenses (general), (4) unmet health care expenses, (5) housing insecurity), and (6) food insecurity.

An overall financial hardship index was calculated as the number of domains each participant reported experiencing (range 0–6). The index was also categorized (4–6: substantial hardship, 2–3: some hardship, 1: a little hardship, and 0: no hardship).

Finally, participants were asked, “Which one of the following would you say best represents your race/ethnicity?” with response options of Latino or Hispanic, American Indian or Alaska Native, Asian, Black or African American, Native Hawaiian or Pacific Islander, White, or multiracial. Latino participants were further stratified by their survey language preference (English vs. Spanish); roughly 87% of Latino participants who took the survey in Spanish also reported having limited English proficiency.

### Statistical analyses

Cochran-Armitage trend tests were used to compare the prevalence of psychological distress (anxiety-depression symptoms, perceived stress, and loneliness-isolation) across financial hardship severity during the pandemic and specific financial hardship domains. When assessing trends across financial hardship severity, psychological distress outcomes were dichotomized (e.g., moderate/severe or mild vs. normal). We used multinomial logistic regression to estimate the associations between financial hardship and psychological distress, adjusting for race-ethnicity, age (categorized as 18–29, 30–39, 40–49, 50–59, 60–69, and ≥70 years old), gender (male, female, and transgender/nonbinary), highest education level (less than high school graduate, high school graduate, some college/vocational school, and college graduate or more), and self-reported physical health (poor/fair or good/very good/excellent).

Using the financial hardship index as the exposure of interest (range 0–6), the same methods were used to estimate the average increase in psychological distress associated with experiencing hardship in one additional domain. To assess the associations between each financial hardship domain and psychological distress, we used multinomial logistic regression, adjusting for the same covariates above, including each domain of financial hardship instead of the composite score. Finally, interaction terms between financial hardship (treated as a continuous variable of the number of financial hardship domains experienced) and race-ethnicity were added to the models described above to assess whether the impact of financial hardship during the pandemic on psychological distress differed across racial-ethnic groups.

All analyses were performed in SAS version 9.4 (SAS, Inc., Cary, NC). All analyses were weighted to produce nationally representative estimates within each racial-ethnic group and counts were rounded for interpretation.

## Results

Sociodemographics of participants, overall and stratified by financial hardship severity, are reported in Supplementary Table S1. Overall, 70.3% of participants reported experiencing some level of financial hardship during the first year of the pandemic (substantial hardship: 21.3%; some hardship: 27.4%; and a little hardship: 21.6%); Spanish-speaking Latino (87.3%) and Native Hawaiian/Pacific Islander (79.2%) adults were most likely to report experiencing hardship, Table 1.

**Table 1.** Prevalence of Financial Hardship During the First Year of the Pandemic, Overall and Stratified by Race/Ethnicity, COVID-19's Unequal Racial Burden Survey, December 2020–Februrary 2021

	Substantial hardship	Some hardship	A little hardship	No hardship	Any hardship
	***N*** (%)	***N*** (%)	***N*** (%)	***N*** (%)	***N*** (%)
Overall	1170 (21.3)	1503 (27.4)	1188 (21.6)	1634 (29.7)	3861 (70.3)
Race/ethnicity					
American Indian/Alaska Native	130 (25.9)	151 (30.2)	104 (20.7)	116 (23.2)	380 (76.1)
Asian	121 (12.1)	207 (20.7)	258 (25.9)	413 (41.4)	586 (58.6)
Black/African American	240 (24.0)	294 (29.5)	229 (22.9)	245 (23.6)	763 (76.4)
Latino	293 (29.3)	341 (34.1)	169 (16.9)	196 (19.6)	801 (80.2)
English-speaking	111 (22.4)	143 (28.8)	109 (22.1)	132 (26.7)	362 (73.0)
Spanish-speaking	182 (36.2)	198 (39.4)	59 (11.7)	64 (12.7)	440 (87.3)
Native Hawaiian/Pacific Islander	152 (30.5)	141 (28.4)	100 (20.2)	103 (20.8)	393 (79.3)
White	131 (13.1)	225 (22.6)	224 (22.4)	419 (41.9)	579 (58.0)
Multiracial	104 (20.9)	143 (28.7)	102 (20.5)	150 (29.9)	350 (70.1)

Financial hardship was measured by counting the number of hardship domains each participant reported experiencing (lost income, debt, unmet expenses, unmet health care expenses, housing insecurity, and food insecurity) during the first year of the pandemic and categorized into categories of substantial (4–6), some (2–3), little (1), and no (0) hardship experienced.

The most common financial hardship domains were debt (57.6%), lost income (44.5%), and unmet expenses (33.7%); the prevalence of each financial hardship domain also varied by race-ethnicity, Supplementary Table S2. Spanish-speaking Latino participants were most likely to report lost income (73.1%), debt (80.7%), and housing insecurity (30.8%). Native Hawaiian/Pacific Islander (20.5%) and American Indian/Alaska Native (20.4%) were more likely to report food insecurity, compared to the other racial-ethnic groups.

The prevalence of anxiety-depression symptoms was 48.5% (moderate/severe: 23.7% and mild: 24.8%), 62.4% of participants reported stress (moderate/severe: 34.3%; mild: 28.1%), and 61.1% of participants reported experiencing loneliness-isolation (very often/fairly often: 21.3% and sometimes/almost never: 39.8%).

**FIG. 1.**
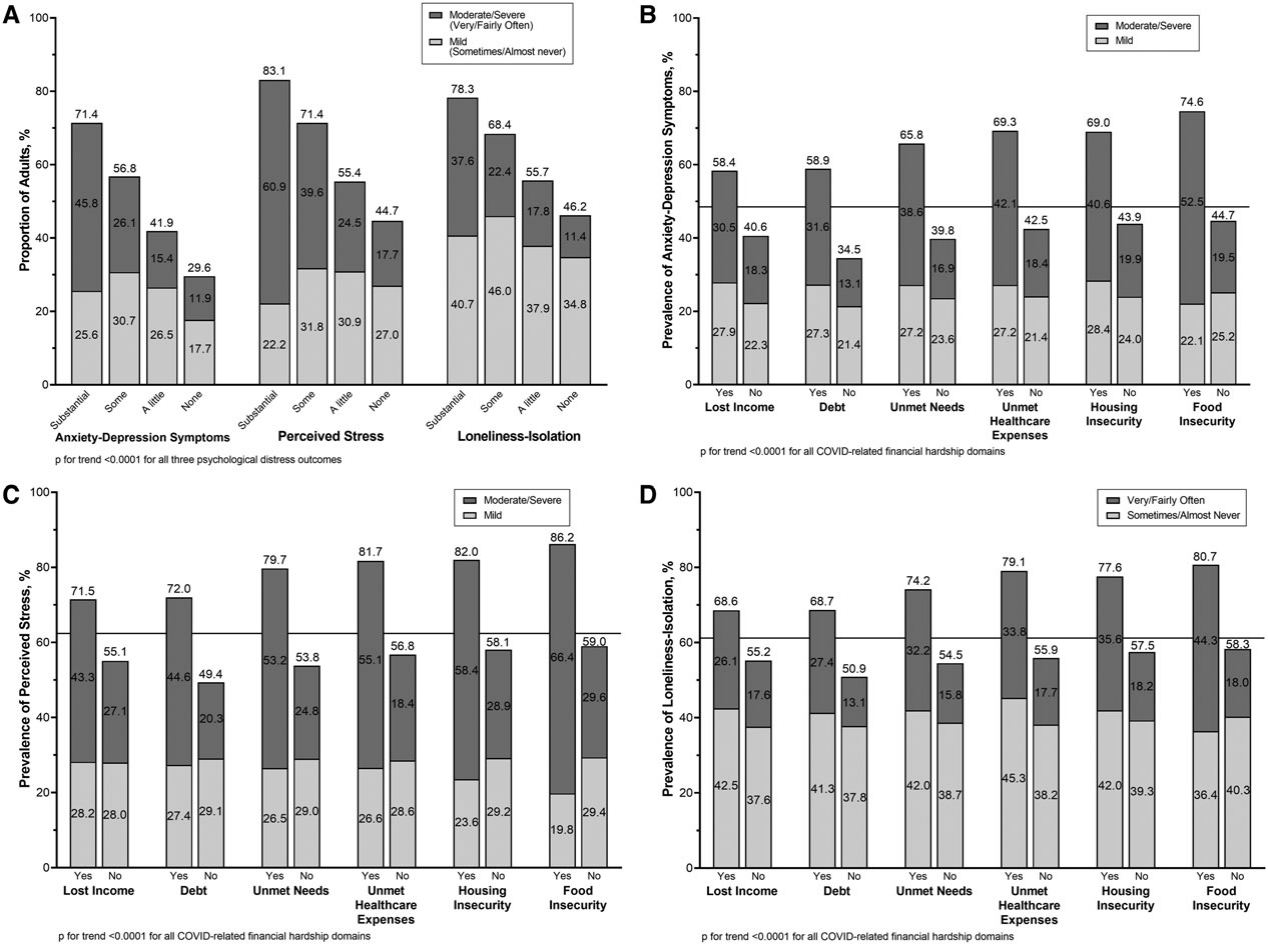
Prevalence of **(A)** psychological distress stratified by experiencing financial hardship during the first year of the pandemic, and the prevalence of **(B)** anxiety-depression symptoms, **(C)** perceived stress, and **(D)** loneliness-isolation by financial hardship domains weighted to be nationally representative within racial-ethnic groups, CURB survey, December 2020–Februrary 2021. CURB, COVID-19's Unequal Racial Burden.

As the amount of financial hardship increased, measured by the number of hardship domains experienced, the prevalence of all three psychological distress measures also increased, *p* for trend <0.0001 for all, Figure 1. For example, 71.4% of adults who experienced substantial hardship had mild or moderate/severe anxiety-depression symptoms, compared to 56.8% among those facing some hardship, 41.9% of those facing a little hardship, and 29.6% facing no hardship. A similar trend was observed across perceived stress and loneliness-isolation.

**FIG. 2.**
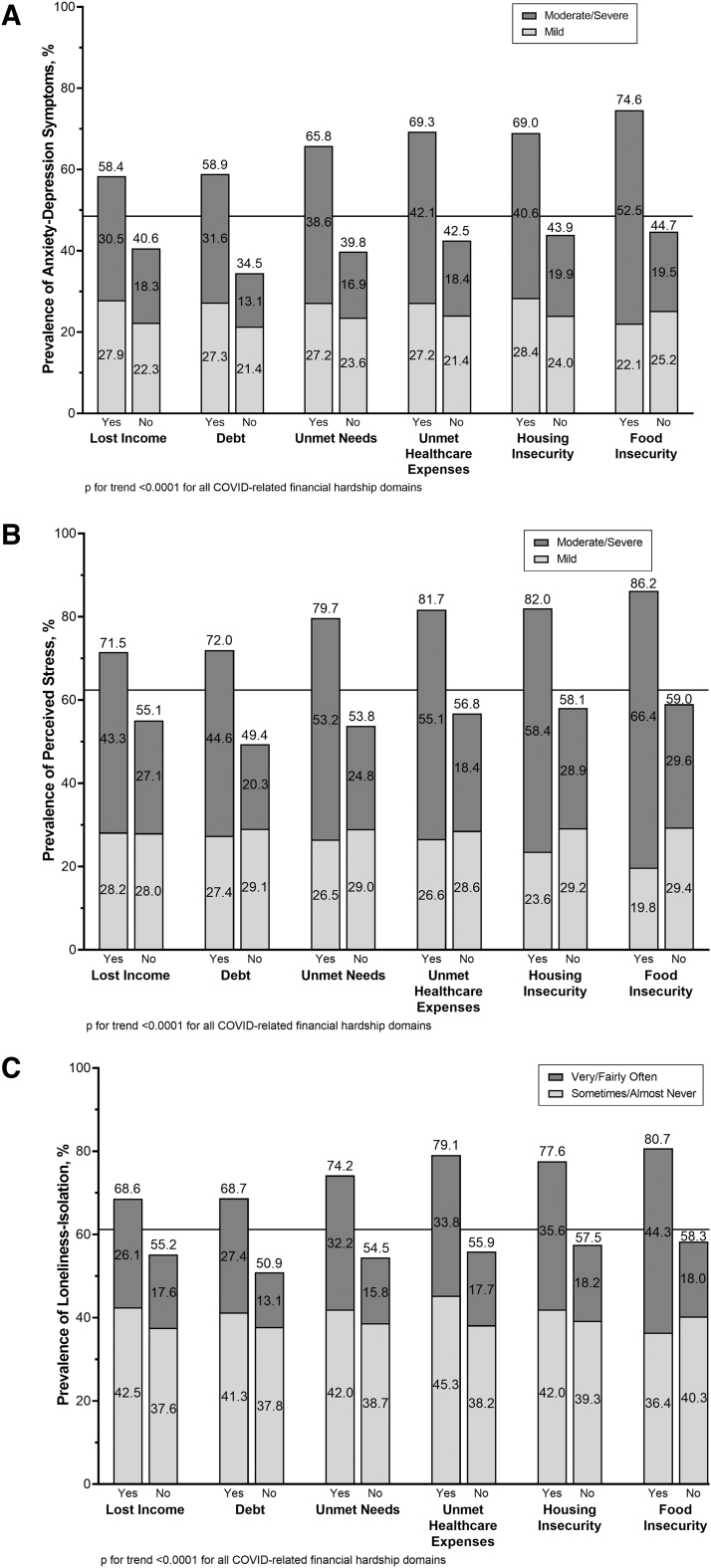
Prevalence of **(A)** anxiety-depression symptoms. **(B)** Perceived stress. **(C)** Loneliness-isolation, stratified by each financial hardship domain, weighted to be nationally representative within racial-ethnic groups, CURB survey, December 2020–Februrary 2021. The horizontal line represents the overall prevalence of that psychological distress measure across all participants (e.g., 48.5% of all participants reported experiencing moderate/severe or mild anxiety-depression symptoms).

Compared to those who did not experience financial hardship, a higher prevalence of anxiety-depression symptoms, perceived stress, and loneliness-isolation was seen among those who experienced financial hardship for all financial hardship domains, p for trend <0.0001 for all, Figures 2A–C. The prevalence of each type of psychological distress varied by financial hardship domain with the largest associations observed for food insecurity, unmet health care expenses, and housing insecurity. For example, for those reporting food insecurity, the prevalence of anxiety-depression symptoms was 74.6%, 86.2% for perceived stress, and 80.7% for loneliness-isolation.

After adjusting for sociodemographics and self-reported physical health, increased levels of financial hardship during the pandemic were still significantly associated with worse psychological distress with a dose–response relationship observed for all three distress measures, Table 2. For example, experiencing some hardship was associated with an almost threefold increase in the odds of both mild and moderate/severe anxiety-depression symptoms (mild: adjusted odds ratio [aOR]=2.75, 95% confidence interval [CI]=2.27–3.32 and moderate/severe: aOR=3.21, 95% CI=2.58–3.98), whereas those who experienced substantial financial hardship were over three times as likely to have mild anxiety-depression symptoms (aOR=3.430, 95% CI=2.74–4.29) and eight times more likely to have moderate/severe anxiety-depression symptoms (aOR=8.15, 95% CI=6.45–10.29), compared to those with no financial hardship.

**Table 2.** Adjusted Associations Between Experiencing Financial Hardship, Compared to No Hardship, Anxiety-Depression Symptoms, Perceived Stress, and Loneliness-Isolation, COVID-19's Unequal Racial Burden Survey, December 2020–Februrary 2021

	Substantial hardship	Some hardship	A little hardship
	aOR (95% CI)^a^	aOR (95% CI)^a^	aOR (95% CI)^a^
Anxiety-depression symptoms^b^			
Moderate/severe	8.15 (6.45–10.29)	3.21 (2.58–3.98)	1.42 (1.12–1.80)
Mild	3.43 (2.74–4.29)	2.75 (2.27–3.32)	1.77 (1.46–2.15)
Perceived stress^c^			
Moderate/severe	10.47 (8.27–13.27)	4.14 (3.38–5.07)	1.60 (1.30–1.98)
Mild	2.92 (2.30–3.72)	2.42 (2.01–2.92)	1.44 (1.20–1.74)
Loneliness-isolation^d^			
Very/fairly often	8.18 (6.37–10.52)	3.36 (2.67–4.23)	1.88 (1.48–2.38)
Sometimes/almost never	3.11 (2.53–3.83)	2.36 (1.99–2.80)	1.35 (1.14–1.60)

All analyses were weighted to be nationally representative within racial-ethnic groups.

Financial hardship was measured by counting the number of hardship domains each participant reported experiencing (lost income, debt, unmet expenses, unmet health care expenses, housing insecurity, and food insecurity) during the first year of the pandemic and categorized into categories substantial (4–6), some (2–3), little (1), and no (0) hardship experienced.

^a^ Adjusted for race-ethnicity, gender, age, highest education level, and self-reported physical health.

^b^ Anxiety-depression was measured with the PHQ-4 and scored as none (0), very mild (1–2), mild (3–5), moderate (6–8), or severe (9–12) and then collapsed to none, very mild/mild, or moderate/severe, due to small numbers in some of the categories.

^c^ Perceived stress was assessed with a 6-item adapted version of the PSS-10; scoring=low (1; reference), mild (1.1–2), or moderate/severe stress (2.1–5).

^d^ Loneliness was assessed with a single item that asks how often in the past month they felt lonely and isolated; scoring=never (1; reference), almost never/sometimes (2–3), or fairly often/very often (4–5).

aOR, adjusted odds ratio; CI, confidence interval; PHQ-4, Patient Health Questionnaire for Depression and Anxiety; PSS-10, Perceived Stress Scale-10.

Similar trends were observed when examining the prevalence and severity of perceived stress and loneliness-isolation, Table 2. On average, experiencing one additional hardship was associated with a roughly 30% increase in mild psychological distress (anxiety-depression symptoms: aOR=1.29, 95% CI=1.23–1.35; perceived stress: aOR=1.30, 95% CI=1.24–1.37; and loneliness-isolation: aOR=1.29, 95% CI=1.24–1.34), and a 60% increase in the odds of moderate/severe psychological distress (anxiety-depression symptoms: aOR=1.60, 95% CI=1.53–1.68; perceived stress: aOR=1.71, 95% CI=1.63–1.79; and loneliness-isolation: aOR=1.57, 95% CI=1.49–1.65), Supplementary Table S3.

Although all hardship domains were associated with all psychological distress measures, there were differences in the magnitude of associations across hardship domains, Table 3. Overall, food insecurity had the strongest association with moderate/severe psychological distress (anxiety-depression symptoms: aOR=1.94, 95% CI=1.52–2.46; perceived stress: aOR=1.99, 95% CI=1.51–2.63; and loneliness-isolation: aOR=1.87, 95% CI=1.43–2.44). Unmet health care expenses were also strongly associated with experiencing moderate/severe anxiety-depression symptoms (aOR=1.94, 95% CI=1.61–2.34), moderate/severe perceived stress (aOR=1.93, 95% CI=1.59–2.36), and feeling lonely-isolated very/fairly often (aOR=1.90, 95% CI=1.54–2.34). Debt had the third strongest association with moderate/severe distress levels (anxiety-depression symptoms: aOR=1.73, 95% CI=1.43–2.09; perceived stress: aOR=1.82, 95% CI=1.52–2.17; and loneliness-isolation: aOR=1.74, 95% CI=1.42–2.12).

**Table 3.** Adjusted Associations Between Financial Hardship Domains, Anxiety-Depression Symptoms, Perceived Stress, and Loneliness, COVID-19's Unequal Racial Burden Survey, December 2020–Februrary 2021

	Lost income^a^	Debt^b^	Unmet expenses^c^	Unmet health care expenses^d^	Housing insecurity^c^	Food insecurity^f^
	aOR (95% CI)^g^	aOR (95% CI)^g^	aOR (95% CI)^g^	aOR (95% CI)^g^	aOR (95% CI)^g^	aOR (95% CI)^g^
Anxiety-depression symptoms^h^						
Moderate/severe	1.26 (1.06–1.48)	1.73 (1.43–2.09)	1.67 (1.37–2.05)	1.94 (1.61–2.34)	1.23 (0.98–1.53)	1.94 (1.52–2.46)
Mild	1.28 (1.10–1.49)	1.45 (1.22–1.71)	1.27 (1.05–1.54)	1.39 (1.16–1.68)	1.30 (1.04–1.61)	0.96 (0.74–1.25)
Perceived stress^i^						
Moderate/severe	1.25 (1.07–1.47)	1.82 (1.52–2.17)	1.94 (1.58–2.37)	1.93 (1.59–2.36)	1.50 (1.18–1.89)	1.99 (1.51–2.63)
Mild	1.20 (1.02–1.40)	1.27 (1.07–1.50)	1.57 (1.28–1.93)	1.55 (1.26–1.91)	1.10 (0.86–1.41)	1.09 (0.81–1.48)
Loneliness-isolation^j^						
Very/fairly often	1.15 (0.97–1.38)	1.74 (1.42–2.12)	1.52 (1.22–1.89)	1.90 (1.54–2.34)	1.46 (1.15–1.86)	1.87 (1.43–2.44)
Sometimes/almost never	1.21 (1.05–1.40)	1.23 (1.05–1.43)	1.27 (1.06–1.53)	1.83 (1.52–2.19)	1.28 (1.03–1.58)	1.01 (0.79–1.30)

^a^ Lost income included loss of job or reduced hours, or loss of work-related income.

^b^ Unmet expenses included not having enough money to meet daily needs or not enough money to pay monthly bills.

^c^ Debt included using up all/most of savings, having no savings before the pandemic, or having gone into debt or increased debt during the pandemic.

^d^ Unmet health care expenses included loss of health insurance, not having enough money to pay for health care, and not having enough money to pay for medications.

^e^ Housing insecurity included not having a regular place to live and not having enough money to pay rent, mortgage, or housing costs.

^f^ Food insecurity included being hungry but did not eat because not enough money for food.

^g^ Adjusted for other financial hardship domains, race-ethnicity, gender, age, highest education level, and self-reported physical health.

^h^ Anxiety-depression was measured with the PHQ-4 and scored as none (0), very mild (1–2), mild (3–5), moderate (6–8), or severe (9–12) and then collapsed to none, very mild/mild, or moderate/severe, due to small numbers in some of the categories.

^i^ Perceived stress was assessed with a 6-item adapted version of the PSS-10; scoring=low (1; reference), mild (1.1–2), or moderate/severe stress (2.1–5).

^j^ Loneliness was assessed with a single item that asks how often in the past month they felt lonely and isolated; scoring=never (1; reference), almost never/sometimes (2–3), or fairly often/very often (4–5).

The association between the financial hardship index and psychological distress was relatively consistent across racial-ethnic groups, *p*≥0.10 for all, Supplementary Table S3.

## Discussion

Using data from a nationally representative cross-sectional survey of a diverse cohort of 5500 adults living in the United States, we found that almost three-quarters of participants experienced some form of financial hardship during the first year of the pandemic. Moreover, as the level of financial hardship increased, the prevalence and severity of psychological distress (anxiety-depression symptoms, perceived stress, and loneliness-isolation) also increased in a dose–response manner.

When broken down by specific financial hardship domains, experiencing food insecurity, unmet health care expenses, and increasing debt had the largest impacts, and lost income alone had the smallest impact, on psychological distress. The impact of financial hardship during the pandemic on psychological distress was also relatively consistent across all racial-ethnic groups; however, given that financial hardship was more commonly experienced by racial-ethnic minorities, the economic effects of the COVID-19 pandemic could still create and exacerbate mental health disparities in the United States.

At least one other study also reported that food insecurity has a profound impact on psychological distress among low-income U.S. adults during the COVID-19 pandemic.^15^ Food insecurity has been shown to be more prevalent in racial-ethnic minorities^16^; however, few studies have examined psychological distress and food security together in historically marginalized populations. To date, research on food insecurity and psychological distress before the pandemic has primarily focused on Black/African American^17^ and Latino^18^; adults. In our nationally representative survey, we found American Indian/Alaska Native and Native Hawaiian/Pacific Islander adults experienced the highest levels of food insecurity.

At least one other study has also found that food insecurity during the COVID-19 pandemic was higher among Native Hawaiian/Pacific Islander adults, compared to White, Asian, and Latino adults, although estimates were similar to those of Black adults; American Indian/Alaska Native adults were not included in the study.^19^ Given that American Indian/Alaska Native and Native Hawaiian/Pacific Islander adults experienced disproportionately higher rates of mental health problems (e.g., post-traumatic stress disorder, depression, suicide) before the pandemic,^20,21^ food insecurity during the pandemic could further exacerbate psychological distress disparities among these groups.

Unmet health care expenses also had a consistently large impact on anxiety-depression symptoms, perceived stress, and loneliness-isolation during the pandemic. Having unmet health care expenses during the pandemic was also more prevalent in racial-ethnic minority groups (American Indian/Alaska Native, Native Hawaiian/Pacific Islander, Black/African American, Spanish-speaking Latino, and multiracial adults), compared to White adults. While this may be the first study to assess the impact of unmet health care expenses on psychological distress during the pandemic, the impact of health care costs and the inability to pay for medications, treatment, and preventative services on psychological distress and mental health before COVID-19 have been well-documented.^22,23^ For example, Medicare beneficiaries with a history of depression have been found to experience greater inability to access care, higher unmet social and economic needs, and worse mental health outcomes.^23^

Historically, economic downturns tend to exacerbate existing socioeconomic inequalities. Although we did not note racial-ethnic differences in the impact of financial hardship on psychological distress, that does not mean the economic effects of the COVID-19 pandemic will not create or exacerbate disparities in psychological distress and mental health. Research has consistently shown that individuals with lower education and without secure employment are more likely to experience financial hardship during economic crises and are more vulnerable to mental health declines.^24–26^ During the Great Recession (2007–2009), individuals in lower-ranked occupations and lower-paid occupations were most vulnerable to becoming unemployed.^24^

Racial-ethnic minorities were overrepresented in the sectors that were hit hardest by the recession, and therefore disproportionally experienced job loss and related economic and mental health-related consequences.^27,28^ Moving forward, alleviating financial hardship caused by the pandemic will continue to be especially critical for racial-ethnic minorities and other groups at higher risk for poor mental health and those that experience disparate outcomes as a result of economic hardships.

This study has a few limitations. First, the survey was administered online, and individuals with limited internet access or familiarity with technology are less likely to participate. While we did match and weigh participants to obtain a nationally representative sample, it is possible that some selection bias exists, especially given the correlation between access to internet, teleworking, and employment opportunities during the COVID-19 pandemic. We also had relatively low response rate from YouGov panel members (20%). It is possible that individuals who were more likely to participate had different experiences of financial hardship and psychological distress during the pandemic, and post-sampling survey weights may not fully account for these differences.

Second, the CURB survey was only administered in English and Spanish (Latino participants only), which means other non-English speaking adults were more likely to be excluded. Since it is currently estimated that 31.9% of Asian adults living in the United States have limited English proficiency,^29^ and at least one study found that Asian adults with limited English proficiency were particularly impacted by COVID-related economic hardships,^30^ our findings may not generalize to all Asians (in our survey, 12.3% of Asian participants reported limited English proficiency). Asian adults are also a very heterogenous group, and it is possible that financial hardship could impact psychological distress differently among different communities. We also did not include Middle Eastern/North African adults in our study, and the potential burden of financial hardship and its impact on psychological distress among this population are still largely unknown.

Finally, while we were able to capture financial hardship across several domains, these measures are not comprehensive, and some participants may have been incorrectly classified as not having experienced financial hardship. For example, we asked if individuals had “lost [their] job or business,” but did not ask about other household or family members, which could also cause hardship. However, we expect this misclassification would bias results toward the null. Future research should assess how family size and makeup may change the impact of financial hardship on mental health, including its potential impact on children and dependents. We were also unable to determine if the financial hardship was caused by the COVID-19 pandemic and COVID-related policies.

Overall, our results provide a detailed understanding of the associations between financial hardship and anxiety-depressive symptoms, perceived stress, and loneliness-isolation during the pandemic. To our knowledge, this is the most in-depth assessment of the effects of financial hardships during the pandemic on psychological distress, and the first to include American Indian/Alaska Native and Native Hawaiian/Pacific Islander adults. Food insecurity and unmet health care expenses were consistently the most strongly associated with severe anxiety-depression symptoms, stress, and loneliness-isolation. Given that racial-ethnic minorities were more likely to experience financial hardship during the COVID-19 pandemic, the long-term economic impacts of the pandemic could create or widen pre-existing disparities in psychological distress in the United States.

Continued advocacy for financial relief efforts like living wages, child tax credits, and hazard pay is not only essential for providing economic assistance to those in need but could also improve individual and community psychological well-being and quality of life. To ensure the effectiveness of food assistance and mental health programs and reduce the impact of food insecurity and unmet health care expenses on psychological distress and well-being, both outreach and access to relevant services and health insurance must remain a priority.

## Supplementary Material

Supplemental data

Supplemental data

Supplemental data

## Abbreviations Used

aOR: adjusted odds ratio
CI: confidence interval
COVID-19: coronavirus disease 2019
COPE: COVID-19 Participant Experience
CURB: COVID-19's Unequal Racial Burden
PHQ-4: Patient Health Questionnaire for Depression and Anxiety
PSS-10: Perceived Stress Scale-10
