# Supplementary material for: Financial Hardship and Psychological Distress During the Pandemic: A Nationally Representative Survey of Major Racial-Ethnic Groups in the United States
Source: Health Equity. 2023 Jul 20;7(1):395–405. doi: 10.1089/heq.2022.0197 (PMC10362911; doi:10.1089/heq.2022.0197)
Supplement: Supplemental data [file Suppl_TableS1.docx]

Supplemental Table 1. Sociodemographics, overall and stratified by financial hardship during the pandemic, CURB survey, December 2020-Februrary 2021.

|  | Overall | Substantial  Hardship | Some  Hardship | A Little  Hardship | No  Hardship |
| --- | --- | --- | --- | --- | --- |
| Total, N | 5,500 | 1,170 | 1,503 | 1,188 | 1,634 |
| Age, years, median (IQR) | 42 (29, 58) | 36 (27, 47) | 40 (29, 55) | 44 (30, 60) | 50 (33, 65) |
| Race/ethnicity, n (%) |  |  |  |  |  |
| American Indian/Alaska Native | 500 (9.1) | 130 (11.1) | 151 (10.0) | 104 (8.7) | 116 (7.1) |
| Asian | 1,000 (18.2) | 121 (10.3) | 207 (13.8) | 258 (21.7) | 413 (25.3) |
| Black/African American | 1,000 (18.2) | 240 (20.5) | 294 (19.6) | 231 (19.4) | 235 (14.4) |
| Latino |  |  |  |  |  |
| English-speaking | 496 (9.0) | 111 (9.5) | 143 (9.5) | 110 (9.3) | 132 (8.1) |
| Spanish-speaking | 504 (9.2) | 182 (15.6) | 198 (13.2) | 59 (5.0) | 64 (3.9) |
| Native Hawaiian/Pacific Islander | 500 (9.1) | 152 (13.0) | 141 (9.4) | 100 (8.4) | 103 (6.3) |
| White | 1,000 (18.2) | 131 (11.2) | 225 (15.0) | 224 (18.8) | 420 (25.7) |
| Multiracial | 500 (9.1) | 104 (8.9) | 143 (9.5) | 102 (8.6) | 150 (9.2) |
| Gender, n (%) |  |  |  |  |  |
| Male | 2,588 (47.1) | 525 (44.9) | 669 (44.5) | 581 (48.9) | 808 (49.7) |
| Female | 2,771 (50.5) | 598 (51.1) | 789 (52.5) | 594 (50.0) | 789 (48.5) |
| Non-binary^b^ or transgender | 133 (2.4) | 47 (4.0) | 45 (3.0) | 12 (1.0) | 30 (1.8) |
| Health insurance, n (%) |  |  |  |  |  |
| Any private | 1,137 (20.8) | 403 (34.7) | 384 (25.7) | 193 (16.3) | 157 (9.7) |
| Public insurance only | 2,384 (43.6) | 288 (24.8) | 515 (34.4) | 556 (46.9) | 1,025 (63.0) |
| Uninsured | 1,953 (35.7) | 471 (40.5) | 596 (39.9) | 436 (36.8) | 445 (27.3) |
| Immigration status, n (%) |  |  |  |  |  |
| US-born citizen | 4,276 (77.8) | 893 (76.4) | 1,119 (74.5) | 964 (81.2) | 1,296 (79.3) |
| Foreign-born citizen\legal resident | 946 (17.2) | 180 (15.4) | 290 (19.3) | 194 (16.3) | 282 (17.3) |
| Undocumented | 275 (5.0) | 96 (8.2) | 93 (6.2) | 30 (2.5) | 56 (3.4) |
| Limited English proficiency^c^, n (%) | 618 (11.2) | 188 (16.1) | 248 (16.5) | 95 (8.0) | 87 (5.3) |
| Education, n (%) |  |  |  |  |  |
| Less than high school | 498 (9.1) | 156 (13.4) | 164 (10.9) | 100 (8.4) | 74 (4.5) |
| High school/GED | 1,791 (32.6) | 442 (37.7) | 507 (33.7) | 392 (33.0) | 451 (27.6) |
| Some college/vocational | 1,690 (30.7) | 412 (35.2) | 476 (31.7) | 345 (29.0) | 457 (28.0) |
| College graduate or more | 1,520 (27.6) | 160 (13.7) | 356 (23.7) | 352 (29.6) | 652 (39.9) |
| Family annual income^d^, n (%) |  |  |  |  |  |
| <$20,000 | 1,095 (22.8) | 404 (37.4) | 367 (27.6) | 220 (21.8) | 104 (7.5) |
| $20,000-$59,999 | 1,921 (40.0) | 485 (44.9) | 608 (45.8) | 355 (35.2) | 469 (33.8) |
| $60,000-$99,999 | 974 (20.3) | 140 (13.0) | 225 (16.9) | 255 (25.3) | 355 (25.6) |
| ≥$100,000 | 818 (17.0) | 51 (4.7) | 129 (9.7) | 179 (17.7) | 459 (33.1) |
| Prefer not to say | 692 | 89 | 174 | 181 | 248 |
| Married^e^, n (%) | 2,551 (46.4) | 467 (39.9) | 686 (45.6) | 516 (43.4) | 882 (54.0) |
| Household configuration, n (%) |  |  |  |  |  |
| Lives alone | 978 (17.8) | 174 (14.9) | 261 (17.4) | 258 (21.7) | 282 (17.3) |
| Adults only | 2,593 (47.1) | 434 (37.1) | 631 (42.0) | 576 (48.5) | 951 (58.2) |
| Single parent and child(ren) | 229 (4.2) | 90 (7.7) | 76 (5.1) | 27 (2.3) | 36 (2.2) |
| Adults and child(ren) | 1,699 (30.9) | 472 (40.3) | 534 (35.6) | 328 (27.6) | 365 (22.3) |
| Census division, n (%) |  |  |  |  |  |
| New England | 160 (2.9) | 17 (1.4) | 52 (3.4) | 39 (3.3) | 52 (3.2) |
| Middle Atlantic | 622 (11.3) | 110 (9.4) | 157 (10.5) | 151 (12.7) | 204 (12.5) |
| East North Central | 554 (10.1) | 105 (8.9) | 153 (10.2) | 111 (9.3) | 185 (11.3) |
| West North Central | 224 (4.1) | 51 (4.4) | 49 (3.3) | 47 (4.0) | 76 (4.6) |
| South Atlantic | 1,070 (19.5) | 204 (17.4) | 286 (19.0) | 230 (19.3) | 351 (21.5) |
| East South Central | 252 (4.6) | 78 (6.7) | 84 (5.6) | 36 (3.0) | 54 (3.3) |
| West South Central | 668 (12.2) | 175 (15.0) | 181 (12.0) | 146 (12.3) | 166 (10.2) |
| Mountain | 555 (10.1) | 137 (11.7) | 174 (11.6) | 102 (8.6) | 138 (8.4) |
| Pacific | 1,394 (25.4) | 293 (25.0) | 367 (24.4) | 326 (27.5) | 408 (24.9) |
| Residence urbanicity, n (%) |  |  |  |  |  |
| Big city | 1,433 (26.8) | 367 (31.7) | 392 (26.5) | 296 (25.8) | 374 (24.1) |
| Smaller city | 1,022 (19.1) | 271 (23.4) | 302 (20.4) | 215 (18.8) | 234 (15.0) |
| Suburban area | 1,664 (31.1) | 237 (20.4) | 412 (27.8) | 397 (34.6) | 619 (39.8) |
| Small town | 659 (12.3) | 171 (14.7) | 187 (12.7) | 129 (11.3) | 172 (11.0) |
| Rural | 566 (10.6) | 114 (9.8) | 186 (12.6) | 110 (9.6) | 156 (10.0) |
| Abbreviations: IQR, interquartile range  ^a^ Financial hardship was measured by counting the number of hardship domains each participant reported experiencing (lost income, debt, unmet expenses, unmet healthcare expenses, housing insecurity, food insecurity) and categorized as substantial (4-6), some (2-3), a little (1), or no hardship (0)  ^b^ Includes individuals who identified as non-binary, gender queer, gender fluid, other, and none  ^c^ Limited English proficiency was defined as speaking English "not at all", "poorly", and "fairly well"  ^d^ Collected by YouGov at enrollment into panel and updated every 6 months; does not account for household size  ^e^ Collected by YouGov at enrollment into panel and updated every 12 months; includes domestic partnership | | | | | |
