# Supplementary material for: Financial Hardship and Psychological Distress During the Pandemic: A Nationally Representative Survey of Major Racial-Ethnic Groups in the United States
Source: Health Equity. 2023 Jul 20;7(1):395–405. doi: 10.1089/heq.2022.0197 (PMC10362911; doi:10.1089/heq.2022.0197)
Supplement: Supplemental data [file Suppl_TableS2.docx]

Supplemental Table 2. Prevalence of financial hardship domains during the pandemic, overall and stratified by race/ethnicity, CURB survey, December 2020-Februrary 2021.

|  | **Lost Income** | **Debt** | **Unmet Expenses** | **Unmet Healthcare Expenses** | **Housing Insecurity** | **Food Insecurity** |
| --- | --- | --- | --- | --- | --- | --- |
|  | **N (%)** | **N (%)** | **N (%)** | **N (%)** | **N (%)** | **N (%)** |
| **Overall** | 2,448 (44.5) | 3,165 (57.6) | 1,854 (33.7) | 1,250 (22.7) | 1,008 (18.3) | 707 (12.9) |
| **Race/ethnicity** |  |  |  |  |  |  |
| American Indian/Alaska Native | 223 (44.6) | 328 (65.5) | 189 (37.8) | 143 (28.6) | 110 (22.0) | 102 (20.4) |
| Asian | 393 (39.3) | 385 (38.5) | 194 (19.4) | 176 (17.6) | 103 (10.3) | 56 (5.6) |
| Black/African American | 428 (42.9) | 643 (64.3) | 419 (41.9) | 247 (24.7) | 215 (21.5) | 157 (15.7) |
| Latino | 579 (57.9) | 728 (72.8) | 449 (44.9) | 259 (25.9) | 247 (24.7) | 128 (12.8) |
| English-speaking | 210 (42.4) | 322 (64.9) | 180 (36.3) | 115 (23.1) | 92 (18.3) | 62 (12.5) |
| Spanish-speaking | 368 (73.1) | 406 (80.7) | 269 (53.3) | 144 (28.6) | 155 (30.8) | 66 (13.1) |
| Native Hawaiian/Pacific Islander | 259 (51.9) | 332 (66.4) | 221 (44.7) | 137 (27.6) | 136 (27.4) | 102 (20.5) |
| White | 349 (34.9) | 464 (46.4) | 226 (22.6) | 162 (16.2) | 109 (10.9) | 90 (9.0) |
| Multiracial | 217 (43.4) | 285 (57.1) | 156 (31.2) | 126 (25.3) | 89 (17.8) | 72 (14.5) |
