# Supplementary material for: Financial Hardship and Psychological Distress During the Pandemic: A Nationally Representative Survey of Major Racial-Ethnic Groups in the United States
Source: Health Equity. 2023 Jul 20;7(1):395–405. doi: 10.1089/heq.2022.0197 (PMC10362911; doi:10.1089/heq.2022.0197)
Supplement: Supplemental data [file Suppl_TableS3.docx]

Supplemental Table 3. Overall and race/ethnicity-specific associations between financial hardship^a^ and psychological distress (anxiety-depression symptoms, perceived stress, loneliness-isolation), CURB survey, December 2020-Februrary 2021.

|  | **Anxiety-Depression Symptoms^b^** | |  | **Perceived Stress^c^** | |  | **Loneliness-Isolation^d^** | |
| --- | --- | --- | --- | --- | --- | --- | --- | --- |
|  | **Moderate/Severe**  **aOR (95% CI)^e^** | **Mild**  **aOR (95% CI)^e^** |  | **Moderate/Severe**  **aOR (95% CI)^e^** | **Mild**  **aOR (95% CI)^e^** |  | **Very/Fairly Often**  **aOR (95% CI)^e^** | **Sometimes/**  **Almost never**  **aOR (95% CI)^e^** |
| **Overall** | 1.60 (1.53-1.68) | 1.29 (1.23-1.35) |  | 1.71 (1.63-1.79) | 1.30 (1.24-1.37) |  | 1.57 (1.49-1.65) | 1.29 (1.24-1.34) |
| **Race/ethnicity** |  |  |  |  |  |  |  |  |
| American Indian/Alaska Native | 1.87 (1.60-2.18) | 1.38 (1.19-1.60) |  | 1.73 (1.48-2.01) | 1.21 (1.03-1.41) |  | 1.66 (1.43-1.94) | 1.27 (1.11-1.45) |
| Asian | 1.72 (1.52-1.93) | 1.41 (1.27-1.57) |  | 1.79 (1.57-2.02) | 1.48 (1.30-1.68) |  | 1.68 (1.48-1.91) | 1.36 (1.22-1.52) |
| Black/African American | 1.60 (1.45-1.77) | 1.34 (1.22-1.48) |  | 1.71 (1.55-1.89) | 1.34 (1.21-1.48) |  | 1.60 (1.44-1.78) | 1.26 (1.16-1.37) |
| Latino |  |  |  |  |  |  |  |  |
| English-speaking | 1.40 (1.19-1.66) | 1.10 (0.97-1.24) |  | 1.52 (1.31-1.76) | 1.22 (1.07-1.39) |  | 1.35 (1.13-1.60) | 1.18 (1.05-1.32) |
| Spanish-speaking | 1.67 (1.44-1.94) | 1.30 (1.13-1.49) |  | 1.68 (1.44-1.95) | 1.26 (1.08-1.46) |  | 1.51 (1.30-1.77) | 1.32 (1.16-1.50) |
| Native Hawaiian/Pacific Islander | 1.43 (1.26-1.63) | 1.19 (1.05-1.35) |  | 1.67 (1.45-1.93) | 1.37 (1.18-1.58) |  | 1.47 (1.28-1.69) | 1.19 (1.06-1.33) |
| White | 1.68 (1.50-1.88) | 1.29 (1.16-1.45) |  | 1.91 (1.67-2.18) | 1.29 (1.13-1.48) |  | 1.76 (1.55-2.00) | 1.46 (1.30-1.64) |
| Multiracial | 1.48 (1.29-1.69) | 1.29 (1.13-1.47) |  | 1.59 (1.36-1.85) | 1.21 (1.02-1.42) |  | 1.48 (1.27-1.73) | 1.33 (1.14-1.53) |
| **P-value for interaction^f^** | 0.095 | |  | 0.230 | |  | 0.245 | |
| Abbreviations: aOR, adjusted odds ratio; CI, confidence interval  ^a^Financial hardship was measured by counting the number of hardship domains each participant reported experiencing (lost income, debt, unmet expenses, unmet healthcare expenses, housing insecurity, food insecurity) and modeled as a continuous, linear variable  ^b^Anxiety-depression was measured with the PHQ-4 and scored as none (0), very mild (1-2), mild (3-5), moderate (6-8) or severe (9-12) and then collapsed to none, very mild/mild, or moderate/severe, due to small numbers in some of the categories  ^c^ Perceived stress was assessed with a 6-item adapted version of the Perceived Stress Scale-10; scoring=low (1; reference), mild (1.1-2), or moderate/severe stress (2.1-5)  ^d^ Loneliness was assessed with a single item that asks how often in the past month they felt lonely and isolated; scoring = never (1; reference), almost never/sometimes (2-3), or fairly often/very often (4-5)  ^e^ Adjusted for race-ethnicity, gender, age, highest education level, and self-reported physical health; interpreted as the average change in psychological distress associated with experiencing financial hardship in one more domain during the pandemic  ^f^P-value from the interaction term between financial hardship index and race-ethnicity in the generalized logistic regression model (df=14) | | | | | | | | |
